# Malignant mesothelioma incidence by nation-wide cancer registry: a population-based study

**DOI:** 10.1186/s12995-016-0127-4

**Published:** 2016-07-26

**Authors:** Kristinn Tomasson, Gunnar Gudmundsson, Haraldur Briem, Vilhjalmur Rafnsson

**Affiliations:** 1 Department of Occupational Medicine, Administration of Occupational Safety and Health, Reykjavik, Iceland; 2 Faculty of Medicine, University of Iceland, Reykjavik, Iceland; 3 Department of Respiratory Medicine and Sleep, Landspitali University Hospital, Fossvogur, 108 Reykjavik, Iceland; 4 Centre for Health Security and Communicable Disease Control, Directorate of Health, Reykjavik, Iceland; 5 Department of Preventive Medicine, University of Iceland, Stapi/ Hringbraut, Reykjavik, Iceland

**Keywords:** Malignant mesothelioma, Cancer registry, Incidence rates, Death registry, Mortality, Asbestos import, Relative risk, Trend, Population

## Abstract

**Background:**

Malignant mesothelioma caused by asbestos exposure has a long latency period. A ban on asbestos use may not be apparent in decreased incidence in the population until after several decades. The aim was to evaluate changes in the incidence of malignant mesothelioma, and the possible impact of the asbestos ban implemented in Iceland in 1983.

**Methods:**

This is a population study on aggregate level; the source of data was the Icelandic Cancer Registry, the National Cause-of-Death Registry, and the National Register. Volume of asbestos import was obtained from Customs Tariff. The import figures reflect fairly accurately the amount used, as there are no mines in the country.

**Results:**

Asbestos import peaked in 1980 at 15.0 kg/capita/year, diminishing to 0.3 kg/capita/year ten years after the ban in 1983, and to zero in the most recent years. Seventy-nine per cent of the cases of malignant mesothelioma were men, and 72 % were of pleural origin. Mesothelioma incidence increased steadily from 1965 to 2014, when it reached 21.4 per million among men, and 5.6 among women. Mortality in 2014 was 22.2 per million among men, and 4.8 among women.

**Conclusion:**

Malignant mesothelioma incidence and mortality increased in the population during the period, despite the ban on asbestos use from 1983. This is in agreement with the long latency time for malignant mesothelioma. In line with the previously high per capita volume of asbestos import, many buildings, equipment, and structures contain asbestos, so there is an on-going risk of asbestos exposure during maintenance, renovations and replacements. It is thus difficult to predict when the incidence of malignant mesothelioma will decrease in the future. During the last ten-year period, the incidence in Iceland was higher than the recently reported incidence in neighbouring countries.

## Background

Malignant mesothelioma is a cancer of mesothelial serosa; its primary location is pleura, peritoneum, pericardium, and tunica vaginalis. Since the association between malignant mesothelioma and asbestos exposure was first described by Wagner and co-workers in 1960 [1], it has been established and recognized that asbestos is the dominant cause of malignant mesothelioma, according to studies from several national mesothelioma registries [2–5]. Today, all types of asbestos, chrysotile, crocidolite, amosite, anthophyllite, tremolite, and actinolite are considered causes of human malignant mesothelioma according to the International Agency for Research on Cancer (IARC) report [6] where epidemiological, experimental, and mechanistic studies have been evaluated. Chrysotile asbestos was the last type to be definitively included as a cause of malignant mesothelioma [7–9]. The time from first exposure to asbestos until diagnosis of malignant mesothelioma is called the induction/latency time. This period can be from 20 to 50 years [10–12], with a shorter latency in the heavily exposed population and longer latency among groups with low-level exposure. In the case of crocidolite exposed cohort the risk of malignant mesothelioma increases from 10–15 years following first exposure and persists for as long as the exposed persons survive, however stops increasing after 30–40 years [13]. The long latency period poses difficulties in occupational studies; however the risk of malignant mesothelioma caused by asbestos is dose-dependent [4, 14, 15], and malignant mesothelioma occurs at very low-level exposure, indeed lower than the eight-hour occupational threshold limit value generally established at 0.1 fibre/ml air, and no safe dose has been found, below which there is no risk of malignant mesothelioma [16].

In addition to asbestos exposure, other casual associations with malignant mesothelioma include radiation treatment for Hodgkin lymphoma and non-Hodgkin lymphoma [17, 18]. Malignant mesothelioma has also been found as a second cancer among testis and prostate cancer patients treated with radiotherapy [19, 20]. At one time, the Simian Virus 40 was thought to play a role in the aetiology of malignant mesothelioma; however, according to IARC, there is inadequate evidence to consider Simian Virus 40 a carcinogen to humans [21]. Erionite is a fibrous mineral belonging to a group of minerals called zeolite and fluoro-edenite another type of fibrous mineral have been associated with malignant mesothelioma in specific areas in Turkey, Mexico and Sicily [22–25]. Erionite is classified as a human carcinogen according to IARC [6], and is widely prevalent in the US [26] and in Iceland [27].

Mortality information on mesothelioma is compiled internationally in the WHO database [28]. Information on the incidence of malignant mesothelioma is commonly obtained from special national malignant mesothelioma registers [2–5], or the cancer registry, Surveillance, Epidemiology, and End Results (SEER) [29]. Where cancer registries and special occupational disease registries can be compared on the national level, the cancer registries show numerical superiority compared with the occupational disease registries, as reported in studies from some Scandinavian countries [30–33]. The setting in Iceland, where the cancer registry was established in 1955 [34], and where there is access to comprehensive population registries as well as general use of a personal identifier, provides an opportunity to study malignant mesothelioma incidence.

The aim of the study was to describe the long-term changes in incidence of malignant mesothelioma up to 2014, and to evaluate the possible impact of the asbestos ban implemented in 1983.

## Methods

This is a population-based observational study and the source of data was the Icelandic Cancer Registry. The registry is nation-wide and includes all cases of cancer since 1955 [34]. The registry has virtually complete coverage, and over 95 % of diagnoses are histologically confirmed [34, 35]. We asked for information from the registry on all cases of cancer with ICD-10 code C45 mesothelioma (the accurate location was read from the decimal figure, pleura, peritoneum, and other and unspecified sites), and ICD-O codes 9050, 9051, 9052, and 9053, mesothelioma, with the behaviour code /3, malignant, primary site. Different versions of the ICD were in use during the operation of the registry, and they were standardized by the registry to ICD-10. Besides the information on the location and morphology of the cases, we obtained information on encrypted personal identification number, gender, age, year and age at diagnosis. The registry does not routinely collect information on occupational or environmental exposure, or survival of the reported cancer cases.

Statistics Iceland maintains the National Cause-of-Death Registry [36]. The registry is nation-wide and includes causes of death according to death certificates. The ICD-10 code C45 was available for 1996–2014.

Three broad categories of exposure to asbestos have been described. The first is for miners and millers of raw asbestos and people employed by manufacturers of asbestos products, such as asbestos cement factories, often associated with heavy exposure. The second category is for workers such as carpenters, plumbers, shipbuilders, machine engineers, and insulators who use asbestos products, and other less-exposed occupations such as seamen, fishermen, and various factory workers. The third category is for groups and individuals with short-term or low-level exposure to asbestos, often non-occupational, including family members of asbestos workers, people living near an asbestos industry, and those dwelling or working in buildings containing asbestos. Asbestos production or mining activities have never occurred in Iceland, so the asbestos exposure in question would be of the second category, involving, for example, the building industry, fishing, and geothermal industry, and of the third category.

Information on the volume of annual import of asbestos and goods containing asbestos was obtained from Statistics Iceland [36], according to Customs Tariff, the Directorate of Customs [37], codes under 2524, 6811, 6812, 6813, and 8708 with mention of asbestos, and corresponding codes from older versions of the Customs Tariffs. The amount of asbestos in the Customs Tariff was declared in metric tons and types of asbestos were not indicated.

To estimate the incidence rates for malignant mesothelioma, the numerator used was the number of cases during ten-year periods 1965–1974, 1975–1984, 1985–1994, 1995–2004, and 2005–2014, with the corresponding population figures of those 15 years of age or older [38] obtained from Statistics Iceland [36]. The ten-year rates were compared with the rate in the first period 1965–1974, calculating relative risk (RR), and 95 % confidence intervals (CI). Similarly, the mortality rates were estimated in eight- and ten-year periods 1996–2004, and 2005–2014.

The incidence rates, 95 % CI of the rates, and statistical analyses were performed using the software packet Epi-Info, and Microsoft Excel 7.

The National Bioethics Committee (VSNb201015040006/03.03) approved the study, and the Data Protection Commission was notified of the study and did not make remarks on the way it was conducted.

## Results

**Fig. 1:**
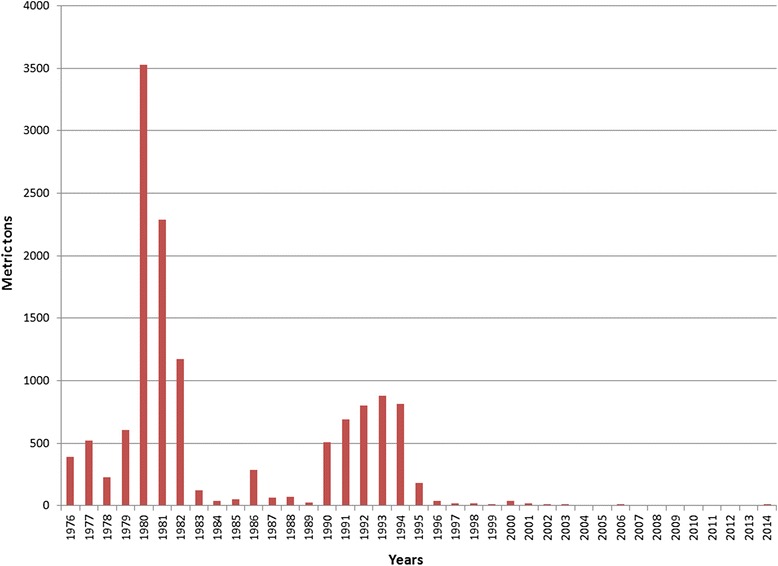
Annual weight of import in metric tons by tariff numbers with mention of asbestos

Information on the annual import of asbestos was available for the period 1976 to 2014, and is shown in Fig. 1. Import volume peaked in the year 1980 at 3500 tons, corresponding to 1500 tons per 100,000 inhabitants, or 15.0 kg/capita/year of asbestos. This huge import coincided with extensive construction of district heating systems, where the main feeding pipes consisted of asbestos cement pipes, and geothermal hot water was piped 20 to 50 km into houses of communities with greater than 10,000 inhabitants. The ban against asbestos use with certain exceptions was implemented in 1983, and that year, import volume decreased considerably, to increase again over a five-year period, and peaking again in 1992 with 800 tons, corresponding to 299 tons per 100,000, or 0.3 kg/capita/year of asbestos. We do not have an explanation for this temporary increase in asbestos import; however, it is clear that this volume involves asbestos cement used in building materials and pipes, or similar articles, as friction material in brake linings and clutches, and fabricated asbestos fibres seldom exceeded 20–30 tons per year in the early period, and did not ever reach an annual volume of one ton during the last ten years of the period.

In table 1, the number of cases of malignant mesothelioma, ten-year annual incidence rates and 95 % confidence intervals, are shown for men and women. Among men, the rates increase steadily through this 50 -year period; however, the changes over the period are not straightforward for women, where the rates are low, and no cases occur in the period 1975–1984.

**Table 1:** Number of malignant mesothelioma cases in ten years periods by gender, annual incidence per million, and 95 % confidence interval (CI)

		1965–1974	1975–1984	1985–1994	1995–2004	2005–2014
Male	Number of cases	1	4	9	19	27
	Incidence per 10^6^	1.4	4.8	9.4	17.6	21.4
	95 % CI	0.1–7.1	1.5–11.6	4.6–17.2	10.9–26.9	14.4–30.7
Female	Number of cases	2	0	3	4	7
	Incidence per 10^6^	2.9	-	3.1	3.7	5.6
	95 % CI	0.5–9.6	-	0.8–8.5	1.2–8.8	2.4–11.0

Table 2 shows age distribution of malignant mesothelioma cases by gender, with the highest number in the age group 70 to 79. In table 3, location and histological diagnosis are shown by gender. In ninety-seven per cent of cases among men, the mesothelioma is located in the pleura, but among women, 56 % is pleura and 44 % is located in peritoneum. Malignant mesothelioma NOS (M-9050/3) is the dominant histological type for both genders.

**Table 2:** Number of malignant mesothelioma cases and deaths by age categories, and gender

		<50	50–59	60–69	70–79	>80	Total
Male	Number of cases	3	8	16	20	13	60
Female	Number of cases	2	1	4	5	4	16
Male	Number deceased	1	5	7	18	11	42
Female	Number deceased	0	0	4	4	2	10

**Table 3:** Number of malignant mesothelioma by sites, gender, and morphology

		M-9050/3	M-9051/3	M-9052/3	M-9053/3	Total
		NOS	Fibrous	Epithelioid	Biphasic	
		n (%)	n (%)	n (%)	n (%)	
Male	Pleura	40 (69.0)	1 (1.7)	14 (24.1)	3 (5.2)	58
	Peritoneum	-	-	1 (100)	-	1
	Other and unspecified sites	1 (100)	-	-	-	1
Female	Pleura	8 (88.9)	-	1 (11.1)	-	9
	Peritoneum	6 (85.7)	-	1 (14.3)	-	7

In table 4, the number of deaths due to malignant mesothelioma according to death certificates, and annual mortality rates is shown by gender. The period available for mortality was 18 years, and no obvious increase over this short observational period and limited volume of data is noted. However, the rates and the RR were high.

**Table 4:** Number of malignant mesothelioma deaths in eight and ten years periods, annual mortality per million per ≥ 15 years (yr), with 95 % confidence interval (CI), and relative risk (RR) and 95 % confidence intervals (CI) during 1996 to 2014, among men and women, first period is the reference

		1996–2004	2005–2014	RR	95 % CI
Male	Number of deaths	14	28		
	Mortality per 10^6^	14.4	22.2	1.54	0.82–3.00
	95 % CI	8.2–23.6	15.0–31.7		
Female	Number of deaths	4	6		
	Mortality per 10^6^	4.1	4.8	1.17	0.32–4.69
	95 % CI	1.3–9.8	1.9–9.9		

Table 5 shows again the number of cases per ten-year periods through 1965 to 2014, and the relative risk, where the first ten-year period, 1965–1974 is the reference. Among men, the relative risk increases over the years, and the trend is statistically significant. The pattern is not so clear among women; however the relative risks increase over the years, but the trend is not statistically significant.

**Table 5:** Number of incident cases of malignant mesothelioma in ten years periods, and relative risk (RR) and 95 % confidence intervals (CI) during 1965 to 2014 among men and women, first period is the reference

		Number of cases	RR	95 % CI
Male	1965–1974	1	Ref.	
	1975–1984	4	3.34	0.37–29.84
	1985–1994	9	6.53	0.83–51.57
	1995–2004	19	12.24	1.64–91.39
	2005–2014	27	14.90	2.03–109.7
	Trend, *p*-value <0.000			
Female	1965–1974	2	Ref.	
	1975–1984	0	-	
	1985-1994	3	1.07	0.18–6.43
	1995–2004	4	1.26	0.23–6.89
	2005–2014	7	1.92	0.40–9.22
	Trend, *p*-value 0.11			

## Discussion

There is still a rising incidence of malignant mesothelioma in Iceland, particularly among men. The increase may be real; however, it may also be partly a reflection of improvement in the diagnoses, and greater watchfulness for the cancer. The ban on asbestos use in 1983 may not have been as influential in Iceland as in neighbouring countries [39]. No case of malignant mesothelioma has ever been reported as an occupational disease in Iceland, so such registration of cases is even less effective than in the other Scandinavian countries [30–33]. The observed incidence of malignant mesothelioma in the last ten-year period is high and consistent with the high incidence rate reported by countries in the global overview. Only Australia, Great Britain, Belgium and the Netherlands had higher rates [12]. This is noteworthy, as there are no asbestos mines and there never has been any asbestos production industry in Iceland. However, the import figures are astonishingly high in relation to the population. In a report on disease burden due to asbestos in European countries, the age-adjusted mortality rate of malignant mesothelioma was highest in Iceland, 25 per million people [40]. That figure is not much higher than the rate 22.2 found in the present study, in spite of some differences in methodologies used to calculate the rates.

In the past the volume of the asbestos import was among the highest per capita in a European study [40], and this was confirmed in the present study. The description of the import of asbestos in the present study shows the same pattern as has been observed in previous studies on asbestos use, where a ban has been established; the national consumption of asbestos has diminished from high per capita use to an almost immeasurably small amount in the past few years [39, 40]. In these recent years, it is the friction material in brake linings and clutches that is still important [41]. However, other sources of asbestos exposure are related to home maintenance and renovation [42], and the maintenance of the asbestos-containing structure of the extensive hot water supply systems in Iceland. During the last 20 years, labour inspection, the Administration for Occupational Safety and Health [43], has been enforcing safe handling of asbestos in maintenance work. According to information from the cancer registry, no cluster of malignant mesothelioma has been identified at time of diagnosis in the areas with known erionite occurrence in the country.

Malignant mesothelioma is not the only cancer attributed to asbestos exposure. There is sufficient evidence that asbestos exposure causes lung, larynx and ovarian cancer, and limited evidence that it causes colorectal, pharynx and stomach cancers [6]. Lung cancer is the most important and is generally more frequent than malignant mesothelioma in asbestos-exposed cohort studies. However, lung cancer caused by asbestos is under-recognized and often confounded by cigarette smoking [44]. Because of the firm association between asbestos exposure and malignant mesothelioma, its incidence or mortality has been taken as an indicator of asbestos exposure in the population. In a recent study of McCormack and co-workers, malignant mesothelioma mortality was used to estimate asbestos-related lung cancer in different populations [45], and smoking habits were taken into account. In the two models used in that study to estimate asbestos-related lung cancer mortality, the proportion of lung cancers that are a consequence of asbestos exposure ranged between 6.6 to 9.3 % in Iceland [45].

The use of the National Register for the source of denominator strengthens the study. Personal identification numbers, which we had access to in encrypted form, are used in the comprehensive population registers in Iceland, and they ensure that we did not double count cases or dead persons. The National Cause-of-Death Registry, the source of information of number of deaths from malignant mesothelioma, has been reported as good in the global evaluation, and was categorized as high quality overall and ranked in the same category as data from 23 developed countries, including the US and the UK [46]. The autopsy rate is about 14 % of all deaths during the study period [36].

The figures from the two sources of information concerning number of incident cases, and number of deaths due to malignant mesothelioma per calendar year or age categories are not in agreement in the present study. This is partly because there is a natural lag between the diagnosis and the death, further more an autopsy study has revealed that about 45 % of male cases of mesothelioma may remain undiagnosed [47], while still another Italian study has shown that the overall concordance between histo-pathological diagnosis and death certification was about 75 % [48].

To the strength of the study we count the use of the Icelandic Cancer Registry for the source of malignant mesothelioma incidence, in which more than 95 % of the diagnoses in the registry are histologically verified [34, 35], and in the present study all cases were with morphological diagnosis.

The relatively high proportion of peritoneal mesotheliomas among females may raise concern of diagnostic error, and as the cases were not histologically reviewed by an expert pathologist for the purpose of this study, we count this as a limitation of the study.

The study material originates from the comprehensive population registries in Iceland, which are considered to be of high quality; however these are from one country only, which may limit the generalizability of the results. The characteristics of the background population are known and the population is homogenous, being 99 % white Caucasian, with uniform financing of the health care system, also of high quality. The country belongs to the developed countries [45]; however, it differs from other European countries in that heavy industry has expanded during the study period, with aluminium and ferrosilicon plants having been set up to harvest abundant electric power resources.

## Conclusion

The incidence rates of malignant mesothelioma seem to be increasing in Iceland, which indicates that there is no reduction yet of the influence of previous asbestos exposure on the population, despite the ban, first implemented in 1983, in an attempt to diminish the use of asbestos. The previous per capita asbestos import rate was among the highest in Europe. The findings of the high incidence and mortality of malignant mesothelioma is in agreement with the long latency for malignant mesothelioma. The incidence during the last ten-year period in the present study is higher than the recently reported incidence in neighbouring Scandinavian countries.

## Abbreviations

CI, confidence intervals; IARC, International Agency for Research on Cancer; ICD-10, International Classification of Diseases, 10th version; ICD-O, International Classification of Diseases for Oncology, Third Edition; RR, relative risk; SEER, Surveillance Epidemiology and End Results; UK, United Kingdom; US, United States
